# Supplementary material for: MetaRibo-Seq measures translation in microbiomes
Source: Nat Commun. 2020 Jun 29;11:3268. doi: 10.1038/s41467-020-17081-z (PMC7324362; doi:10.1038/s41467-020-17081-z)
Supplement: Supplementary file 10 — Supplementary Data 7 [file 41467_2020_17081_MOESM10_ESM.zip › File2/Confidence_High_Taxonomy/157231_out.krona.html]

Javascript must be enabled to view this page.

members
magnitude
magnitudeUnassigned
count
unassigned
taxon
rank

157231\_out

5

superkingdom
2
5

1224
5
phylum

class
5
28211

356
5
order

family
5

SRS013687\_contig\_number\_25462SRS023914\_contig\_number\_16948SRS053356\_contig\_number\_44834SRS098571\_contig\_number\_38515SRS1041145\_contig\_number\_37926
45401
